# D-galactose supplementation in individuals with PMM2-CDG: results of a multicenter, open label, prospective pilot clinical trial

**DOI:** 10.1186/s13023-020-01609-z

**Published:** 2021-03-20

**Authors:** Peter Witters, Hans Andersson, Jaak Jaeken, Laura Tseng, Clara D. M. van Karnebeek, Dirk J. Lefeber, David Cassiman, Eva Morava

**Affiliations:** 1 Metabolic Center, Department of Pediatrics, University Hospitals Leuven, Herestraat 49, 3000 Leuven, Belgium; 2 Department of Development and Regeneration, KU Leuven, Leuven, Belgium; 3 Hayward Genetics Center, Tulane University School of Medicine, New Orleans, LA USA; 4 Departments of Pediatrics, Emma Children’s Hospital, Amsterdam, The Netherlands; 5 Departments of Pediatrics, Emma Children’s Hospital, Amsterdam University Medical Centers, Amsterdam, The Netherlands; 6 Department of Pediatrics, Radboud Centre for Mitochondrial Medicine, Radboud University Medical Centre, Nijmegen, The Netherlands; 7 Department of Neurology, Translational Metabolic Laboratory, Donders Institute for Brain, Cognition, and Behavior, Radboudumc, Nijmegen, The Netherlands; 8 Metabolic Center, University Hospitals Leuven, Leuven, Belgium; 9 Department of Clinical Genomics, Mayo Clinic, Rochester, USA

**Keywords:** PMM2-CDG, D-galactose, Glycosylation, Congenital disorder of glycosylation (CDG), Nijmegen pediatric CDG rating scale (NPCRS)

## Abstract

PMM2-CDG is the most prevalent congenital disorder of glycosylation (CDG) with only symptomatic therapy. Some CDG have been successfully treated with D-galactose. We performed an open-label pilot trial with D-galactose in 9 PMM2-CDG patients. Overall, there was no significant improvement but some milder patients did show positive clinical changes; also there was a trend toward improved glycosylation. Larger placebo-controlled studies are required to determine whether D-galactose could be used as supportive treatment in PMM2-CDG patients.

*Trial registration* ClinicalTrials.gov Identifier: NCT02955264. Registered 4 November 2016, https://clinicaltrials.gov/ct2/show/NCT02955264

## Introduction

Phosphomannomutase 2-congenital disorder of glycosylation (PMM2-CDG; OMIM: 601,785) is the most common CDG, with more than 1000 reported patients. This multi-system disease typically presents with neurological involvement (intellectual disability, hypotonia, muscle weakness, neuropathy and ataxia) in combination with liver involvement, endocrine disturbances (hypothyroidism, growth failure, hypogonadotropic hypogonadism, hypoglycemia), clotting factor abnormalities (disturbed hemostatic balance of both pro- and anticoagulants), gastrointestinal disease, etc. [1]. There is 20% mortality observed in the first four years followed by a relatively stable disease course [2]. Currently, only symptomatic or supportive therapy is available [3–5].

Nutritional intervention with oral D-galactose (D-gal) has shown beneficial effects in certain CDG, associated with abnormal glycosylation and hypogalactosylation. Oral D-gal supplementation showed biochemical and clinical benefit in TMEM165-CDG, SLC35A2-CDG and SLC39A8-CDG [4–6]. In addition, a CDG caused by biallelic pathogenic variants in *PGM1* is highly responsive to D-gal [7, 8]. As galactose is known to increase nucleotide sugar levels, specifically UDP-galactose and UDP-glucose, in vitro in fibroblasts derived from individuals with and without CDG. [7], we hypothesized that D-gal supplementation and increasing the UDP-hexose pool, could be of benefit in CDG, even without a clear galactosylation defect. Previously, in vitro studies have shown that several different CDG type I cell lines increase glycosylated ICAM1 expression under galactose treatment, including a few cell lines of PMM2-CDG patients. Additionally, galactose also regulates the activity of different galactosyltransferases [6], and galactose could act as a chaperone, similar to its use in Fabry disease [9], as with enzymes with sugar substrates, sugars often funciton as chaperones.

We performed an observational prospective multicenter pilot study and evaluated the clinical and biochemical evolution of 9 patients with PMM2-CDG, taking oral D-gal supplementation for an 18-week period. The study was open label and without placebo control.

## Patients and methods

### Galactose and nijmegen pediatric CDG rating scale (NPCRS)

Participants were evaluated at three clinical sites using the same observational protocol (Tulane University Medical Center, University of Leuven and Amsterdam University Medical Centers) as part of an observational trial (Tulane IRB protocol #517,339, ClinicalTrials.gov NCT02955264; Leuven ethics committee S59698; AMC METC NL61943.018.17). All patients were confirmed patients with PMM2-CDG based on abnormal glycosylation studies and molecular analysis (see Table 1).

**Table 1:** Patient demographics and genetics

	Age at start (y)	gender	Pathogenic variant 1	Pathogenic variant 2	Reference (previous publications with the patient)
1	6	F	p.I120C	p.G228C	PMID: 28425223
			c.359 T > C	c.682G > T	
2	4	M	p.R141H	p.P113L	PMID: 10801058
			c.442G > A	c.338C > T	
3	5	F	p.S47L	p.Q33P	
			c.140C > T	c.98A > C	
4	4	F	p.R141H	p.F183S	
			c.422G > A	c.548 T > C	
5	17	M	p.R141H	p.F119L	PMID: 30293989
			c.422G > A	c.357C > A	
6	15	M	p.D188G	p.V231M	PMID: 30293989
			c.563A > G	c.691G > A	
7	19	M	p.T237R	p.C241S	PMID: 30293989
			c.710C > G	c.722G > C	
8	28	F	p.R141H	p.T81S	PMID: 17694350
			c.422G > A	c.422G > A	
9	2	M	p.R141H	p.F119L	
			c.422G > A	c.357C > A	

The variants shown are described using the NM_000303.2 transcript reference sequence

D-Galactose intake was increased in a stepwise fashion over the 18 week study period per 6 weeks from 0.5 g/kg per day, to 1.0 g/kg per day and eventually 1.5 g/kg per day to minimize gastrointestinal and metabolic side effects. The maximum daily dose of galactose any patient received was 50 g (this amount is within recommended daily intake) and has been demonstrated to be safe [10] previously in CDG patients [8]. Participants were instructed to continue their regular diet.

The Nijmegen Pediatric CDG rating scale (NPCRS) was assessed at the time of inclusion and at the end of the trial. This score comprises three sections (subscores): current clinical function, system specific involvement and current clinical assessment. A lower score indicates a less severe disease [2]. Additionally, serum sialotransferrin isoforms, as a parameter for glycosylation, were monitored. Transferrin was immunopurified from 10 µL of plasma and analyzed by QTOF mass spectrometry (MS) as previously described [11]. Results of asialo- and monosialotransferrin are expressed as percentage of disialotransferrin values.

### Statistics

SPSS 22 for windows (SPSS Inc., Chicago, IL, USA) were used for statistical analysis. All results were expressed as means ± standard deviation. For differences between repeated measurements in the same individual the Wilcoxon signed rank test was used. A p-value less than 0.05 was considered as statistically significant.

#### Results

Nine PMM2-CDG patients participated in this study (4 female, 5 male). Age at inclusion was 11.1 ± 8.5 years (for details and genetics see Table 1).

**Fig. 1:**
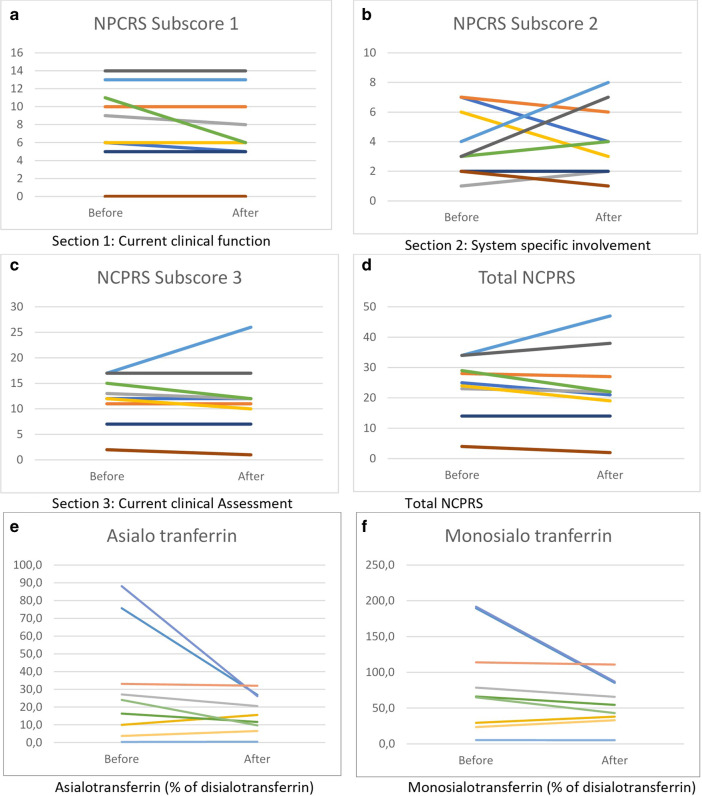
Response to Galactose treatment. Response to escalating dose of oral D-galactose supplementation in nine patients carrying different biallelic pathogenic variants in *PMM2* on the NPCRS score (**a**)–(**d**) and on glycosylation (**e**), (**f**)

Interestingly, the total NCPRS scores improved for 5 patients (see Fig. 1), but due to worsening of two patients the total score didn’t show a statistically significant improvement (score before 23.9 ± 9.6, versus after 23.6 ± 13, *p* = 0.44).There was no significant improvement in the current clinical function (NPCRS subscore 1, Fig. 1) within the total group of 9 patients (score before 8.2 ± 4.4, versus after 7.4 ± 4.4 for the whole group, where a lower score indicates improvement, *p* = 0.10), the system specific involvement (subscore 2 before treatment 3.9 ± 2.3, versus after treatment 4.1 ± 2.4, *p* = 0.77) or in the current clinical assessment (score before 11.8 ± 4.8, versus after 12 ± 6.8, *p* = 0.49).

Nevertheless, in one of the patients (patient 1) significant improvement in quality of life was reported, because of less frequent infections, a growth spurt and fewer episodes of hypoglycemia (NPCRS decreasing from 25 to 21). This effect was noticeable after 6 weeks. Another patient (patient 4) reported a subjective improvement in motor skills, which was not effectively captured in the NPCRS score system and might be part of a more benign disease course in PMM2-CDG (NCPRS from 24 to 19).This effect was only reported at the end of the trial. Also noteworthy are the improvements of one patient (patient 8) with a very mild PMM2-CDG. After 6 weeks, she reported improvement in motor skills as a decrease in titubation (possibility to drink from a cup without spilling, eating soup, improved use of handheld devices, smoother speech, objectively recorded improvement in handwriting), decreased ataxia (less bumping into walls and objects) and increased general activity level. Her NPCRS decreased from 4 to 2. Importantly, after stopping D-gal supplementation her progress reversed. Upon restarting D-gal a similar improvement was seen again.

### Adverse events

There were only gastrointestinal adverse events. Six patients had mild gastrointestinal involvement (mild constipation or unexplained vomiting or diarrhea < 1/week) before starting D-gal. One patient had an increase in PMM2-CDG related diarrhea for which treatment with galactose was temporarily stopped (< 2 weeks).Another patient had a disappearance of all gastrointestinal complaints as the constipation resolved and the polyethylene-glycol preparation was stopped.

### Biochemical variation

As analyzed over the group of 9 patients, the asialotransferrin/disialotransferrin ratio improved from 30.9 ± 31 to 16.6 ± 10.5, which was however not statistically significant (*p* = 0.11). Nevertheless, there was a trend for improvement in monosialotransferrin/disialotransferrin ratio from 84.9 ± 68.2 to 58.1 ± 32.6 (*p* = 0.06).

There was no apparent correlation of the overall biochemical trend with clinical improvements in individual patients.

## Discussion

Overall, in the whole group of nine PMM2-CDG patients there was no statistically significant improvement observed with oral D-gal supplementation. When evaluated as a group, there was no biochemical improvement in the glycosylation profile or detectable clinical improvement in the different sections. Our two patients with the highest NCPRS did not respond at all. Both had profound central nervous system involvement with pyramidal signs. This is probably related to the role of glycosylation in brain development and is as such unlikely to respond to improvement in glycosylation.

Importantly, a few patients did report improvement in different aspects of their clinical symptoms and the mildest patient has experienced objectively measurable clinical changes in neurologic symptoms. Although none of these improvements were captured in a multi-item score as the NPCRS, this did lead to a significant increase in the quality of life of this patient. NCPRS certainly lacks sensitivity in some domains (for example, evolution in writing skills, titubation, decrease in number of infections etc.) as seen in some patients. Nevertheless, in our retrospective natural history study NCPRS has been shown to be stable in untreated patients [2]. Prospective natural history studies are currently being conducted.

D-gal supplementation is safe in numerous CDG [6–8] and in focal segmental glomerulosclerosis [10]. It is interesting to note that in a population with a multisystem disease in which gastrointestinal disease (constipation, diarrhea, failure to thrive, protein losing enteropathy [2, 3]) is an important part, galactose is clinically well tolerated. Indeed, there was no deterioration in GI function as measured by NPCRS.

Larger placebo-controlled, double blind studies are required to determine whether D-gal supplementation can be used as symptomatic treatment in a subset of milder PMM2-CDG patients. Given the lack of reliable, prospectively evaluated biomarkers, clinical findings (such as NCPRS) and CDG validated quality of life outcome measures are preferable outcome measures.

## Data Availability

All generated data is available within the manuscript.

## Abbreviations

CDG: Congenital disorder of glycosylation
PMM2-CDG: Phosphomannomutase2-congenital disorder of glycosylation
D-gal: D-galactose
NPCRS: Nijmegen Pediatric CDG rating scale
MS: Mass spectrometry
